# Medical Literature in the South

**Published:** 1882-12-20

**Authors:** 


### *MEDICAL LITERATURE IN THE SOUTH—OUR FRIENDS DON'T WRITE ENOUGH.*

The rapid advances in the Medical Profession in this country has been due, in a very large degree, to Medical Journalism. Previous to the late war we had to admit that in the Southern section of the Union, we failed, in some measure, to keep up our row, and it is only in the last few years that the profession in the South has evinced or practiced a tithe of the interest in Medical Literature that is due to the ability and talents of its members, or to those who, with little or no profit, were engaged in the self-sacrificing and arduous duties of Journalism. And not yet can it be said that Southern physicians are alive to the duties and responsibilities that rest upon them as medical men in regard to the progress of Medical Literature in their own section. Every progressive and intelligent practitioner is in duty bound to sustain his home Journals and contribute something to the Medical Literature of his own section. Southern practitioners have certainly the ability, but unfortunately they lack the spirit and the energy to write for the Journals. We trust that our readers will take this remark kindly, and that hereafter we shall more frequently receive practical communications for the Original Department of our Journal. To those who have written for us in the past, we are thankful Let your communications be short and practical.

